# Consumer perception of marbling and beef quality during purchase and consumer preferences for degree of doneness

**DOI:** 10.5713/ab.23.0003

**Published:** 2023-05-02

**Authors:** Hakan Benli, Duygu Gecgel Yildiz

**Affiliations:** 1 Department of Food Engineering, Cukurova University, Adana 01330, Turkey

**Keywords:** Beef Quality, Degree of Doneness, Marbling, Purchasing Preference

## Abstract

**Objective:**

Understanding consumer perception of meat quality in developing countries is an important issue since consumer perception of quality could be highly variable. In the current study, consumers’ purchasing preferences affected by marbling and perception of quality were evaluated in a survey study. Furthermore, consumers’ preferences for degree of doneness were investigated using both survey and consumer panel studies.

**Methods:**

The study was carried out in two phases. Firstly, a survey was conducted in Adana Province, Turkey to collect data related to the attributes affecting consumers’ meat purchase decision and consumers’ degree of doneness preferences. In the second phase, boneless ribeye was used to investigate consumers’ degree of doneness preferences in a consumer panel. In addition, proximate analyses of the samples were conducted.

**Results:**

The survey study using pictures of marbling illustrations indicated that higher degrees of marbling might be considered too fatty to be purchased by consumers. Consumers’ perceptions regarding the relationship between marbling and beef quality further indicated that marbling might not be acknowledged as a cue of a higher quality meat. Nevertheless, the results of the importance of some attributes related to intrinsic and extrinsic quality cues showed that consumers were looking for the cues that indicated not only quality but also safety of the meat during meat purchase. The results of both survey and consumer panel studies revealed that consumers might prefer higher degrees of doneness while consuming meat since a majority of the consumers’ preference of degree of doneness was at least well done.

**Conclusion:**

This study revealed that consumer purchasing preferences might vary between countries regarding marbling and perception of quality. Furthermore, higher degrees of doneness could be the preference of these consumers. Thus, further studies are needed to increase consumer satisfaction in these countries.

## INTRODUCTION

People and nations have historically associated the meat consumption with social and/or economic prestige. A positive correlation was indicated between a nation’s economic development and per capita meat consumption. In addition, people tend to increase the amount of meat consumption and demand higher quality meat when they improve their social or economic status [1]. Crops and livestock products data obtained from the Food and Agriculture Organization (FAO) showed that aggregate meat production quantity increased from 179,487 thousand tons in 1990 to 337,180 thousand tons in 2022 in the world [2]. Similarly, Food Balances data collected by FAO indicated increases in aggregate per capita meat consumption from 33.45 kg to 43.22 kg between 1990 and 2013 (the data were collected prior to 2013 with old methodology and population) and from 41.54 kg to 43.16 kg between 2010 and 2019 in the world [3,4]. Meat consumption will likely continue to increase particularly in the developing countries due to the population growth and income increases. However, meat purchasing and eating decisions are directly related to perception of meat by the consumers [5]. Consumers demand higher quality food items in addition to healthy, safe, and enjoyable foods although the perception of quality is highly variable across cultures, societies, and individuals [6]. Since the main purpose of the determination of meat quality is to supply acceptable meat products to meet consumer demand [7], it is important for the meat industry to understand consumers’ perception of the quality in the developing countries to overcome the challenges to maintain their market share and further increase their meat sales in those countries.

Although trained descriptive sensory attributes including tenderness, juiciness and flavor are considered as indicators of consumer acceptability of meat products, consumer sensory studies are also important and widely used to understand consumer preference for meat products [7]. Three basic categories of quality attributes have been defined in literature including search attributes, experience attributes and credence attributes. Search attributes, known also as quality cues, are used by consumers to a make choice among different alternatives of foods during a purchase. There are two types of quality cues including intrinsic and extrinsic cues. While intrinsic cues are related to inherent visible characteristics of a product, extrinsic cues define the information which is not directly related to physical characteristics of the product. Intrinsic and extrinsic cues are used by the consumers to form quality expectations to predict eating quality of a food during consumption. In terms of beef, it was reported that color, marbling, and fat levels were among the most influential intrinsic cues whereas origin and place of purchase were noted among the most significant extrinsic cues. Furthermore, experience attribute is associated with the meat quality that was experienced during consumption including taste, tenderness, juiciness, and flavor. On the other hand, credence attribute cannot be determined even on consumption, but consumers must develop some trust in others for information like safety of the meat and animal welfare [6,8].

The consumer acceptance of beef is directly related with the sensory attributes including tenderness, juiciness, and flavor although tenderness is indicated as one of the most important factors affecting consumers’ overall liking [7,9]. In this regard, marbling level and cooking end point temperature of steaks are considered among the major factors for perceived tenderness during consumption [7,9,10]. There are no known studies in the literature regarding consumers’ marbling and cooking end point preferences related to the perception of quality in Turkey. Understanding consumers’ perception of beef quality could be beneficial for the meat industry to overcome challenges that are specific to developing countries. Thus, the objectives of this study were to collect data related to consumers’ purchasing preferences based on degree of marbling and perception of the quality, the attributes affecting consumers’ meat purchase decisions and consumers’ cooking endpoint temperature preferences by conducting a survey. In addition, consumers’ preferences related to degree of doneness were also investigated by conducting a consumer panel using two whole pieces of boneless ribeye (high and standard quality defined by the producer) that were available in the retail market.

## MATERIALS AND METHODS

The study was carried out in two phases. In the first phase, the data was obtained from a survey conducted in Adana Province (districts of Seyhan, Yuregir, Cukurova), Turkey. In the second phase, two whole pieces of boneless ribeye (one high quality and one standard quality defined by the manufacturer) obtained from healthy 16 to 18 months old male Charolais steers. Then, both were used to investigate the consumers’ preferences related to the degree of doneness.

The procedures of the survey study and consumer panel evaluations were reviewed and approved by the Cukurova University Institutional Review Board (IRB protocol no: 04.09.2015/45).

### Survey study

A questionnaire was used to collect data related to the attributes affecting consumers’ meat purchase decisions and consumers’ cooking endpoint temperature preferences. The sample size (*n* = 384) was determined with following formula [11–16]:


Eq. (1) $$n=t^{2}\;[1+(0.02)\;(b-1)]\times pq/e^{2}$$

where the sample size was represented by *n*, the significance level is represented by *t* (assumed to be 95% with the table value of 1.96), the stage of sampling is represented by *b* (which is equal to 1), the probability of the examined situation occurring is represented by *p* (*p* = 0.5 was used for the absence of preliminary information related to the criteria affecting consumers’ meat purchase decisions), the probability of the examined situation not occurring is represented by *q* (*q* = 1–*p*), and the accepted error is represented by *e* (assumed to be 5%). When *b* equal to 1, the Eq. (1) was converted to the following equation:


$$\begin{array}{l} n=(t^{2}\times pq)/e^{2}\\ n=(1.96^{2}\times 0.5\times 0.5)/0.05^{2}=384 \end{array}$$

The survey was conducted outside the selected supermarkets selling beef products. The questionnaire was prepared as an intercept survey designed to be completed in a few minutes by participating customers [16]. The questions were designed to determine (a) consumers’ purchasing preferences related to the marbling degrees by showing them the pictures of marbling illustrations of North American Meat Processors Association [17] and their general knowledge related to relationship between marbling and meat quality (b) consumers’ perception of beef quality related to some intrinsic and extrinsic quality cues during the meat purchase [8,18] and (c) consumers’ consumption preferences related to the degree of doneness by showing them the pictures of degree of doneness illustrations of North American Meat Processors Association [17].

### Consumer panel

In the second phase, the aim of the study was to investigate the consumers’ preferences related to the degree of doneness using two whole pieces of boneless ribeye (high quality and standard quality defined by the manufacturer) that were available in the retail market. In addition, it was also investigated that whether any differences could be detected by the consumers between two whole pieces of boneless ribeye that were cooked to same degree of doneness (71°C, 77°C, and 82°C). The whole boneless ribeye samples were transported to the laboratory after obtained from the supplier within 45 minutes in an insulated container and stored at refrigerated conditions. The samples were firstly subjected to proximate analyses to determine moisture, ash, fat, and protein content. Then, the boneless ribeye samples were cut into 2.54 cm thick steaks.

Panelists were recruited from three different districts of Adana, Turkey. A total of 103 untrained consumer panelists participated in a total of three sessions completed in same day with 40 participants from Seyhan district, 31 participants from Yuregir district and 32 participants from Cukurova district. The samples were cooked on electrical grill (Grill Comfort; Tefal, Rumilly, France). The internal temperature of each sample was continuously monitored using a handheld thermometer (Type K Thermometer; Verth, Taipei, Taiwan) during the cooking. The samples were cooked to internal temperatures of 71°C (medium), 77°C (well done) and 82°C (very well done) [17,19].

The cooked samples were subjected to a simple ranking test to determine the most preferred sample [20]. 1 cm^2^×2.5 cm pieces were cut from cooked steaks and served for consumers. Both high quality and standard quality boneless ribeye were cooked to three degrees of doneness (medium, 71°C; well done, 77°C; very well done, 82°C) and served to panelists in random order separately. The panelists were asked to rank the samples from the most preferred to the least preferred (1 to 3). In addition, a paired comparison (two-sided) test was used to determine consumers’ preference for the quality grade after cooking samples to three different degrees of doneness (medium, 71°C, well done, 77°C, very well done, 82°C). Cooked boneless ribeye samples were served to panelists in pairs. The panelists were asked to choose the preferred sample between standard quality and high quality.

### Proximate analyses

The oven (Memmert; Universal Oven Tech., Schwabach, Germany) drying method was used to determine moisture contents of the samples at 100°C for 18 h. All the organic matter was ashed in a muffle furnace (PLF 130/45; Protherm, Ankara, Turkey) at 600°C to determine ash contents of the samples. While Soxhlet extraction method was used to determine fat content, the Kjeldahl method was used to determine crude protein content of the samples [21,22].

### Statistical analyses

In the first phase of the study, the data collected from the survey were subjected to Chi-square test, binomial test or Kruskal Wallis test using SPSS software version 20 (IBM SPSS Statistics, Armonk, NY, USA). A simple ranking test was used to determine consumers’ preference for the degree of doneness in sensory evaluation. The data were analyzed with calculating rank sums for each sample. Then a Friedman-type rank test was performed. The nonparametric analogue to Fisher’s least significant difference for rank sums was calculated to further evaluate the samples differed significantly. In addition, a paired comparison (two-sided) test was used to determine consumers’ preference for the quality grade at three different degrees of doneness [20].

## RESULTS AND DISCUSSION

The data regarding education and income levels of meat consumers participated in the study are presented in Table 1. Among the consumers surveyed in the current study, 13.0%, 7.3%, 22.4%, and 19.8% had elementary school, middle school, high school, and associate degree, respectively while 24.7%, 9.6%, and 3.1% of them had bachelor’s degree, master’s degree, and doctoral degree. Turkish Statistical Institute reported that 19%, 19%, and 26% of Turkish people over 15 years old had elementary school, middle school and high school degrees, respectively while 18%, 2%, and 0.4% had bachelor’s degree (including associate degree), master’s degree and doctoral degree in 2021 [23]. Although demographic data indicated that overall the participants had a higher education level than the national average this could be due to the fact that the survey was only conducted outside the selected supermarkets selling beef products.

In addition, the average monthly incomes of the consumers were classified in three categories. Among the 384 consumers, 39.3% indicated having a middle income while 30.5% and 30.2% reported having high and low income levels, respectively. People tend to increase the amount of meat consumption and their demand for higher quality meat when their social or economic status increase [1]. Similarly, Senturk [24] reported that between 2003 and 2013 Turkish consumers increased their demand for the foods from animal origins depending on the increase in their income. In the current study, 69.8% of the participants indicated having a middle- or high-income level which could be an indication of the potential future demands for the meat with higher quantity and/or quality in Turkey as a developing country.

### Purchasing preferences based on degree of marbling

**Figure 1:**
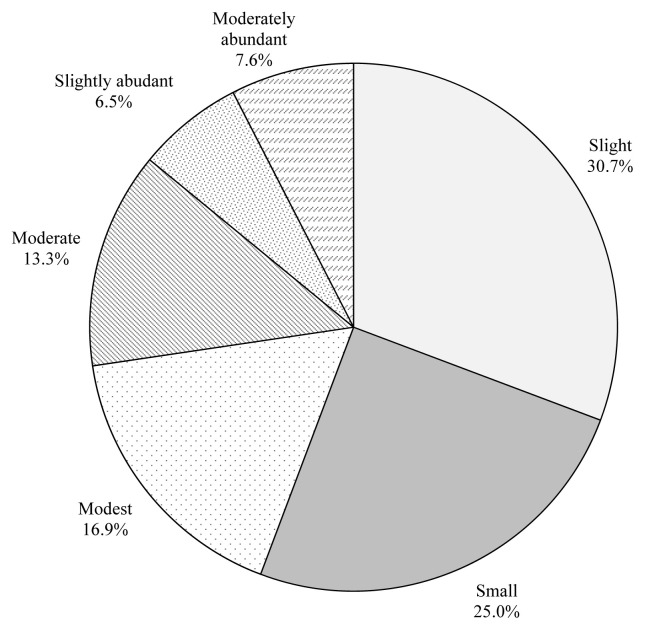
Consumers’ purchasing preferences based on degrees of marbling (%).

The data was collected by showing pictures of marbling illustrations (moderately abundant, slightly abundant, moderate, modest, small, and slight) to the consumers and asking them to indicate their purchasing preference (Figure 1). In the current study, 30.7% of the participants indicated that slight was their purchasing preference while 25% of the participants indicated that small was their purchasing preference. In addition, 16.9% and 13.3% of the participants indicated that modest and moderate were their purchasing preferences, respectively. Furthermore only 6.5% and 7.6% of the total consumers surveyed in the current study reported that slightly abundant and moderately abundant were their purchasing preferences, respectively. There were significant differences (p<0.05; χ^2^ = 107.125; df = 5) among the consumers’ preferences for marbling degree. Consumers use some intrinsic and extrinsic quality cues including marbling, leanness, color, packaging and price etc. to make a buying decision at the point of purchase [25]. Historically, tenderness and juiciness of meat were associated with a pleasant consumption experience [26]. Although health-conscious consumers often preferred low-fat meat to the palatability, consumers perceived the meat juicier when the amount of fat increased. Some of the fat present was released in the first bite or during chewing, thereby stimulating the salivary glands. Thus the meat with higher fat content was perceived as juicier [27]. Savell and Cross [26] developed the “window of acceptability” to show the overall relationship between increased intramuscular fat (marbling) and palatability. They stated that meat with intramuscular fat content of 3% to 7.3% was acceptable. In addition, quality grades of beef carcasses are used to determine eating characteristics including tenderness and palatability of the product. Assignment of a specific quality grade to a carcass requires evaluation of sex characteristics, maturity, the quality of the lean muscle and the degree of marbling [17]. Furthermore, marbling affects the juiciness, tenderness, flavor and appearance of the meat [27]. In the current study, the total preference rate of visuals with slight and small marbling was determined as 55.7% while the total preference rate of visuals with slightly abundant and moderately abundant marbling was determined as 14.1%. These results indicated that the higher degrees of marbling might be considered too fatty for purchasing by the consumers.

### Perceptions regarding marbling and beef quality

**Figure 2:**
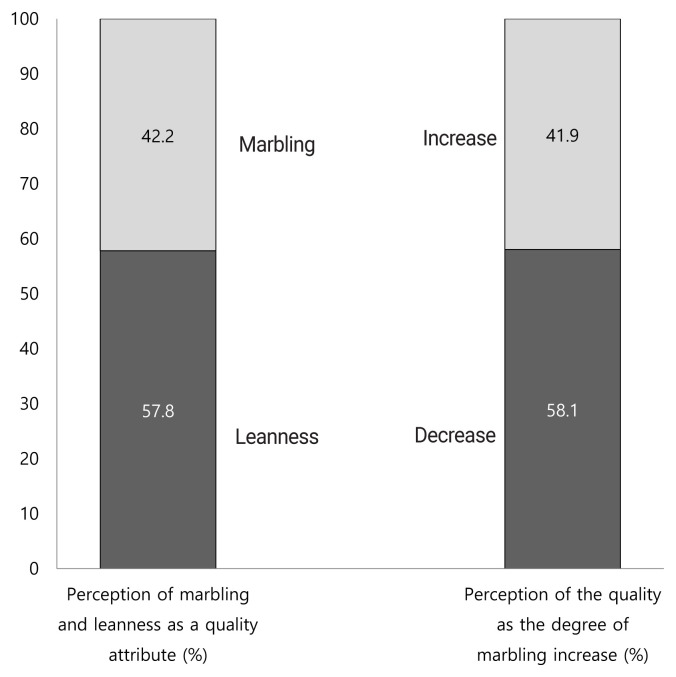
Consumers’ perceptions regarding the relationship between marbling and beef quality.

Furthermore, consumers’ perceptions were also questioned regarding the relationship between marbling and beef quality (Figure 2). Firstly, the participants were asked whether they consider marbling or leanness as a quality attribute for beef. There was a significant difference between consumers’ perception for the leanness and marbling as a quality attribute (p<0.05; χ^2^ = 9.375; df = 1). While 57.8% of the participants indicated that leanness was a quality attribute, 42.2% of the participants indicated that marbling was a quality attribute. In addition, the consumers were also asked how the quality of beef would change when the degree of marbling increased. There was a significant difference between consumers’ interpretations on how the degree of marbling would affect the quality of beef (p<0.05; χ^2^ = 10.010; df = 1). Although 58.1% of the participants indicated that the quality of beef would decrease as the degree of marbling increase, 41.9% of the participants indicated that the quality of beef would increase. While 42.2% of consumers considered marbling as a quality attribute, 41.9% of them stated that higher degree of marbling would affect the quality of beef positively. Nevertheless, the overall preference rate of the pictures of marbling illustrations with moderately abundant (7.6%) and slightly abundant (6.5%) was 14.1%. In addition, 58.1% of the consumers indicated a negative correlation between degree of marbling and beef quality. Even though some participants stated that they cared about intramuscular fat as a quality criterion, they might not have a clear understanding of what marbling was. In fact, the percentage of those who considered leanness as a quality attribute was 57.8%. This agreed with the overall preference rate of the pictures of marbling illustrations with small and slight (55.7%). In general, the results indicated that the higher degrees of marbling might be perceived as too fatty by the consumers and might not be acknowledged as a cue for a higher quality meat.

### Importance of intrinsic and extrinsic quality cues for purchasing

**Figure 3:**
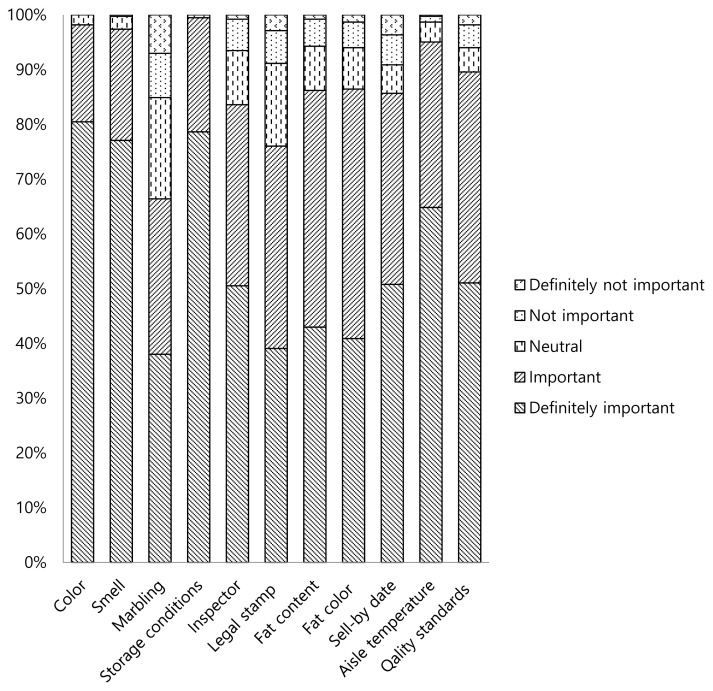
The importance of some attributes (color, smell, marbling, storage conditions, inspector, legal stamp, fat content, fat color, use-by date, the temperature of fresh meat aisle and the compliance with quality standards) for the consumers related to intrinsic and extrinsic quality cues during meat purchase.

A questionnaire was designed using a Likert Scale of 1 to 5 (1 “definitely not important”, 2 “not important”, 3 “neutral”, 4 “important”, 5 “definitely important”) to collect data of importance of some attributes (color, smell, marbling, storage conditions, the presence of inspector, the presence of legal stamp, fat content, fat color, use-by date, the temperature of fresh meat aisle and the compliance with quality standards) related to intrinsic and extrinsic quality cues for the consumers during meat purchase (Figure 3).

Color was indicated as definitely important by 80.5% of the participants while 17.7% and 1.8% of the participants indicated that color was important and neutral, respectively. One of the most important quality attribute of meat was color which also used as an indication of eating quality and safety of meat by consumers [8]. The bright color of meat was associated with the freshness of meat for predicting the quality [5]. Furthermore, the bright red color was defined by most consumers as a desirable meat color and an important factor for purchasing decision due to probable perception of the discoloration as a spoilage indicator [28]. In addition, Droval et al [29] reported that the main quality attributes of meat include appearance, texture, juiciness and flavor and the initial selection of meat is mostly related to the appearance during purchase. Likewise, Arenas de Moreno et al [28] reported that the intrinsic attributes of meat including color, tenderness, juiciness, smell, flavor, and freshness were considered important for the purchasing decision for the most of Venezuelan consumers. Similarly, 98.2% of the consumers indicated that color was definitely important or important during meat purchase in the current study.

Smell was indicated as definitely important by 77.1% of the participants while 20.3%, 2.3%, and 0.3% of the participants indicated that smell was important, neutral, and not important, respectively. Smell was among the experience attributes that mostly related to the safety [8]. In Turkey, meat can be displayed unpackaged in supermarkets. Any spoilage odors generating from the meat might be detected by consumers and could be discouraging during meat purchasing. Becker et al [30] reported that smell was considered either very important or quite important by over 90% of the consumers in Germany. Likewise, 97.4% of the consumers indicated that smell was definitely important or important in the current study. Conversely, Seko et al [5] indicated that consumers were guided by extrinsic quality cues (the salesperson’s expertise, price and quality of the service) during purchasing dibiterie meat while intrinsic quality cues including color and smell of fresh meat had lower concern in Dakar, Senegal.

Marbling was indicated as definitely important by 38% of the participants while 28.4%, 18.5%, 8.1%, and 7.0% of the participants indicated that marbling was important, neutral, not important, and definitely not important, respectively. Marbling was associated with increased beef eating quality [31]. Savell and Cross [26] stated that meat with intramuscular fat content of 3% to 7.3% was acceptable (window of acceptability) for most consumers. However, they also indicated that the palatability decreased significantly with decreases in the fat content if the amount of intramuscular fat was less than 3%. When the fat content exceeded 7.3%, health-conscious consumers defined those meats as too fatty due to the apparent fat content. In addition, Verbeke et al [32] reported that most of European consumers considered lean beef as the healthiest meat. Furthermore, Australian consumers preferred learner meat and avoided visible fat due to health reasons while Asian consumers distinguished intramuscular fat (marbling) from the subcutaneous fat and intramuscular fat was associated with a premium eating experience [33]. Although a total of 66.4% of consumers indicated that marbling definitely important and important during meat purchase, only 14.1% of the consumers preferred the visuals with slightly abundant and moderately abundant marbling in the present study. Similarly, 58.1% of the consumers indicated a negative correlation between degree of marbling and beef quality. These results confirmed that majority of the Turkish consumers might not have a clear understanding of the marbling and they perceived the meat with higher degrees of marbling as too fatty for purchasing. These results were also supported by the importance of fat content and fat color (Figure 3) for consumers during purchasing. Fat content and fat color were indicated by 86.2% and 86.5% of the consumers, respectively as definitely important or important which showed that consumers were very concern about fat and fat related indicators.

Storage conditions were indicated as definitely important by 79% of the participants while 21% of the participants indicated that storage conditions were important. The presence of an inspector was indicated as definitely important by 50% of the participants while 33%, 10%, 6%, and 1% of the participants indicated that the presence of an inspector was important, neutral, not important and definitely not important, respectively. The presence of the legal stamp on carcasses was indicated as definitely important by 39% of the participants while 37%, 15%, 6%, and 3% of the participants indicated that the presence of the legal stamp on carcasses was important, neutral, not important and definitely not important, respectively. Fat content of beef was indicated as definitely important by 43% of the participants while 43%, 8%, 5%, and 1% of the participants indicated that fat content of beef was important, neutral, not important and definitely not important, respectively. Fat color was indicated as definitely important by 41% of the participants while 46%, 7%, 5%, and 1% of the participants indicated that fat color was important, neutral, not important and definitely not important, respectively. Use-by date was indicated as definitely important by 51% of the participants while 35%, 5%, 5% and 4% of the participants indicated that use-by date was important, neutral, not important and definitely not important, respectively. The temperature of fresh meat aisle was indicated as definitely important by 65% of the participants while 30%, 4%, and 1% of the participants indicated that the temperature of fresh meat aisle was important, neutral and not important, respectively. The compliance with quality standards was indicated as definitely important by 51% of the participants while 39%, 4%, 4% and 2% of the participants indicated that the compliance with quality standards was important, neutral, not important and definitely not important, respectively. Becker et al [30] reported that extrinsic cues may play an important role for quality selection at the point of purchase and some of the extrinsic and intrinsic cues could be used by consumers to assess the safety of meat. Similarly, intrinsic cues (color, smell, tenderness, flavor, freshness, and juiciness) and extrinsic cues (aging, hygiene, origin, breed, and animal feeding information) were important for Venezuelan beef consumers in making a buying decision [28]. In contrary, Senegalese consumers were reported to be guided by extrinsic quality cues (the salesperson’s expertise, price, and quality of the service) rather than intrinsic quality cues (color and smell of fresh meat) during purchasing dibiterie meat [5]. In the current study, most of the consumers considered storage conditions, inspector, legal stamp, use-by date, the temperature of fresh meat aisle and the compliance with quality standards definitely important or important during meat purchase. These results indicated that consumers were looking for the cues that indicated not only quality of meat but also safety of the meat at the point of purchase.

### Preferences for degree of doneness

**Figure 4:**
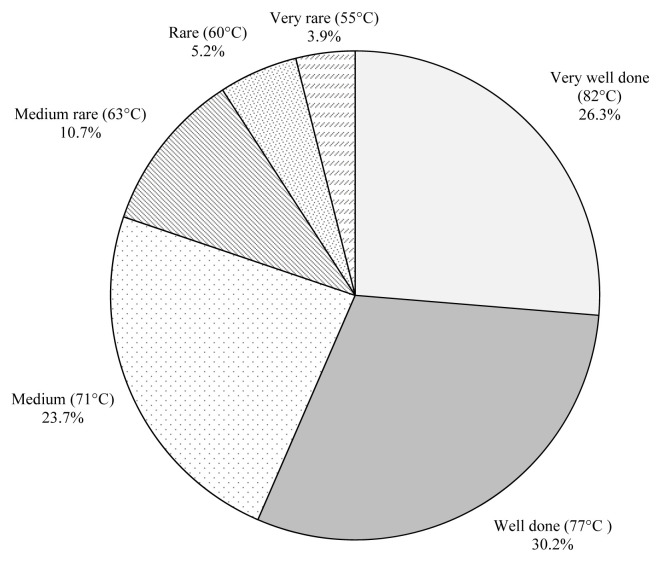
Consumers’ preferences for degree of doneness.

The participants were asked to indicate their preference for degree of doneness (very well done, 82°C; well done, 77°C; medium, 71°C; medium rare, 63°C; rare, 60°C; and very rare, 55°C) of beef steak (Figure 4). In this study, 26% and 30% of the participants indicated that very well done and well done were their degrees of doneness preferences, respectively while 24% of the participants indicated that medium was their degree of doneness preference for the consumption of beef. Only, 11%, 5% and 4% of the participants indicated that their degree of doneness preferences were medium rare, rare and very rare, respectively. There were significant differences (p<0.05; χ^2^ = 151.063; df = 5) among consumers’ degree of doneness preferences. The total preference rate of visuals with very well done and well done was 56% while the total preference rate of visuals with medium rare, rare and very rare was 20%. Lorenzen et al [10] reported that steaks cooked to lower temperatures were tenderer than those cooked to higher temperature due to having higher scores for liking of tenderness, higher scores for liking of juiciness and lower Warner-Bratzler shear values. However, they found no differences in overall liking or liking of flavor among the steaks cooked to different end-point temperatures (very rare, 55°C; rare, 60°C; medium rare, 63°C; medium, 71°C; well done, 77°C; and very well done, 82°C). Reicks et al [34] conducted a study in three different cities including Phoenix, AZ; Baltimore, MD/Washington DC; Lubbock, TX to represent the United States consumers. Among the consumers, 3.4% preferred rare (cool-red center), 26.6% preferred medium rare (warm-red center), 30% preferred medium (pink throughout), 30% preferred medium well (thin line of pink) and 9.9% preferred well done for beef degree of doneness. Conversely, 56% of Turkish consumers preferred well done or very well done while only 9.9% of the U.S. consumer indicated that they preferred well done.

### Preference for degree of doneness - consumer panel

In the second phase of the study, whole boneless ribeye available in the retail markets were used to investigate the consumers’ preferences related to the degree of doneness. These pieces of whole boneless ribeye represented meat samples that could be purchased by the consumers without any additional effort and classified by the manufacturer as “standard quality” and “high quality”. In Turkish markets, meat is offered to customers without any information related the quality grade of the meat during purchase. For most customers, the quality grade is not one of the determining factors for the meat prices since the meat prices are not determined in relation to the quality grade. However, the manufacturers are grading the carcasses to sell higher quality meat to their special customers for higher prices. Thus, we simply asked a local manufacturer for a high quality and a standard quality whole boneless ribeye. The manufacturer supplied us with two whole boneless ribeye samples obtained from healthy 16 to 18 months old male Charolais steers.

Table 2 presents results of the proximate analyses of boneless ribeye samples including moisture, ash, fat, and protein obtained from high quality and standard quality carcasses. There were no statistical differences between the boneless ribeye samples classified as high quality and standard quality for ash, fat, and protein values. However, the moisture values of the samples were statistically different (p<0.05). In addition, chemical lipid values of boneless ribeye samples in the current study were in the range of Low Choice to Top Choice since Low Choice steaks were reported to contain 4% to 5% of chemical lipid with a small degree of marbling and Top Choice steaks were reported to contain 6% to 7% of chemical lipid with modest and moderate degrees of marbling [26,27]

**Figure 5:**
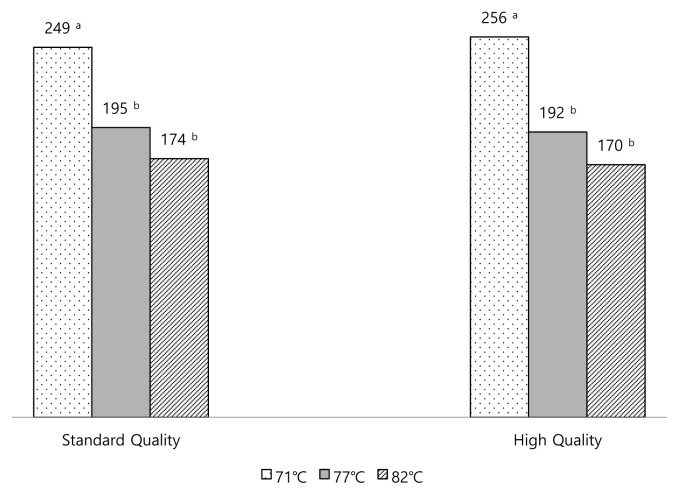
The rank sum values of standard quality and high-quality samples cooked to three different degrees of doneness. The samples were ranked from the most preferred sample to the least preferred sample (1 to 3). ^a,b^ Values with different superscript letters within each quality grade are significantly different (p<0.05).

A simple ranking test was used to determine consumers’ preference for the degree of doneness (Figure 5). Both high quality and standard quality boneless ribeye samples were cooked separately to three different degrees of doneness (medium, 71°C; well done, 77°C; very well done, 82°C) and served to panelists separately in random order. The panelists were asked to rank the samples from the most preferred to the least preferred sample (1 to 3). There were significant differences among the rank sums of standard quality samples cooked to three different degrees of doneness (p<0.05). The rank sums of samples were 249 for medium, 195 for well done and 174 for very well done. Similarly, there were significant differences among the rank sums of high-quality samples cooked to three different degrees of doneness (p< 0.05). The rank sums of samples were 256 for medium, 192 for well done and 170 for very well done. Furthermore, multiple comparison tests for the rank sums indicated that the samples cooked to medium were preferred the least, and the samples cooked to well done and very well done were preferred the most for both standard quality and high-quality samples. The results of the simple ranking test seemed to agree with the results of the survey study that indicated that the total preference rate of visuals with very well done and well done was 56%. Conversely, Lorenzen et al [10] studied the effects of cooking beef steaks to six end point temperatures from very rare (55°C) to up to very well done (82°C). Although cooking steaks to lower end point temperatures were liked by consumers for tenderness and juiciness, the end point temperature had no effect on overall liking. Similarly, beef eating satisfaction was reported that greatly affected by degree of doneness. When cooking end point temperature increased, beef cuts reported to get tougher, less juicy and overall liking decreased [35]. Furthermore, Drey et al [36] reported that increased degree of doneness caused similar negative impact on consumer ratings regardless of marbling level. Contradictory to the literature our results indicated that Turkish consumers might prefer higher levels of degree of doneness even though the tenderness and juiciness were affected negatively. Although marbling level and degree of doneness were important factors for higher consumers’ satisfaction, consumers’ preferences for lower marbling degrees and higher degrees of doneness in some countries would create challenges for the meat industry to satisfy these consumers. For this purpose, a study conducted by Benli and Tokgoz [37] indicated that meat cuts might need to be tenderized to overcome the difficulties due to the raw material quality to satisfy the consumers.

### Preference for quality grades at different degrees of doneness – consumer panel

**Figure 6:**
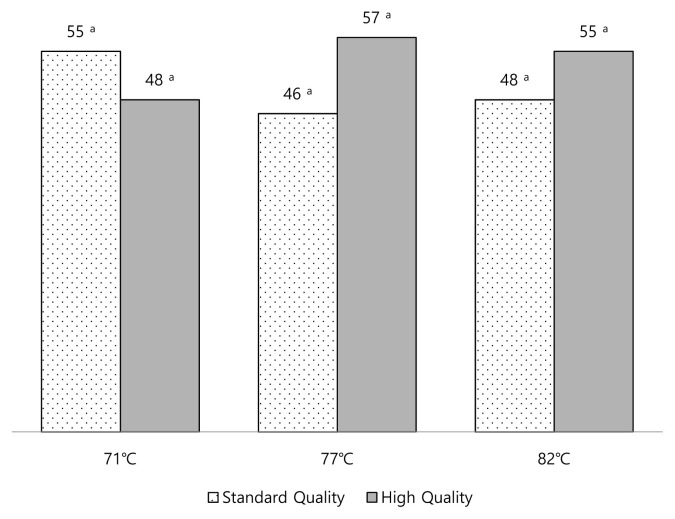
The total number of quality grades chosen by panelists for each degree of doneness. ^a^ Values with different superscript letters within each degree of doneness are significantly different (p<0.05).

A paired comparison (two-sided) test was used to determine consumers’ preferences for the quality grade after cooking samples to three different degrees of doneness. Both high quality and standard quality boneless ribeye samples were cooked separately to specific degrees of doneness including medium (71°C), well done (77°C), very well done (82°C) and served to panelists in pairs. The panelists were asked to choose the preferred sample between standard quality and high quality samples (Figure 6). There were no significant differences among the paired samples at any degree of doneness. In this study we simple asked from a meat producer for a high quality and standard quality whole boneless ribeye that were normally supplied to their customers. Overall, our results and experience indicated that it was difficult to obtain high quality meat in Turkish market even after paying for it if you were not one of the producers’ privileged customers. It seemed that consumers are still at the mercy of the butchers or suppliers to obtain high quality meat from the market. Consumers must develop some personal relationship (regularly buying meat from the same retailer, being a friend of the butcher etc.) to get better quality meat in regular basis. In addition, these results also indicated that the suppliers might be aware of consumers’ choice of the lower marbling levels due to their concerns about the fatty meat.

## CONCLUSION

There are no known studies in literature related to marbling or degree of doneness preferences for Turkish consumers. The current study indicated that higher degrees of marbling might be considered too fatty by most consumers at the point of purchase in Turkey. In addition, consumers were interested in cues related to not only quality of meat but also safety of the meat during purchase. Furthermore, it is well known in literature that degree of doneness influences meat palatability and cooking higher degree of doneness has a negative impact on overall liking. Conversely, majority of the consumers indicated that their preference of degree of doneness was at least well done and above in both survey and consumer panel studies in the current study.

In conclusion, even though quality grading is an important part of meat price determination in some developed countries, in other countries higher graded meats may not be a choice of consumers due to the different preferences related to marbling level. In addition, consumers would prefer a higher degree of doneness while consuming their meat cuts at home or in restaurants in some countries. This could create some problems for retail sellers or restaurants since consumer satisfaction would be lower due to effects of higher degree of doneness preferences. Thus, further studies are needed to overcome challenges related to the preferences including lower degrees of marbling and higher degrees of doneness to increase consumer satisfaction.

## Figures and Tables

**Table 1:** Education and income levels of meat consumers in Adana region (n = 384)

Variable	Categories	Frequency	Percentage (%)
Education level	Elementary School	50	13.0
	Middle School	28	7.3
	High School	86	22.4
	Associate Degree	76	19.8
	Bachelor’s Degree	95	24.7
	Master’s Degree	37	9.6
	Doctoral Degree	12	3.1
Income level^1)^	1,500 ($497) or less	116	30.2
	1,500 ($497) – 3,000 ($993)	151	39.3
	3,000 ($993) or more	117	30.5

1) Average monthly income in Turkish Lira (TL). Average exchange rate in 2016: 1 USD = 3.02 TL.

**Table 2:** Proximate analyses result of boneless ribeye samples defined as high quality and standard quality

Proximate analyses^1)^	High quality	Standard quality
Moisture (%)	69.57±0.56^b^	72.17±1.30^a^
Ash (%)	0.97±0.04^a^	1.01±0.03^a^
Fat (%)	6.36±1.99^a^	5.01±1.51^a^
Protein (%)	19.87±0.66^a^	22.40±1.50^a^

1) The differences between the values (mean±standard deviation) indicated with different letters on the same row are significant (p<0.05).
